# Analecta

**Published:** 1842-02-26

**Authors:** 


### ANALECTA.

*On the performance of Tracheotomy in the last stage of Croup.* By M. Petel, of Cateau.—The object of this paper is to recommend the performance of the operation even in the last stage of the disease; a period when all other remedies are invariably useless. M. Petel has adopted it in six cases, in three of which the patients recovered, though five days had elapsed in one instance and ten in the other from the outbreak of the disease, and both patients were almost *in articulo mortis.* In every case, warm water was thrown into the trachea after the operation several times, always with the effect of facilitating the discharge of mucosities. In one of the cases followed by recovery, a solution of 20 centigrammes of nitrate of silver to 4 grammes of water (about gr. iij. to ℥j.) was dropped into the air-passages; in the other calomel was blown into the trachea. The solution of nitrate of silver was likewise employed in two of the cases which terminated fatally. The subject of the fifth observation had been suffering from croup for eight days when tracheotomy was performed; the patient's life was saved by it, the wound in the trachea healed, and she survived six weeks after the operation, when she died suddenly in the night.

In performing the operation, M. Petel considers it to be inexpedient to delay opening the trachea on account of the hæmorrhage, providing the patient seems in a state of impending suffocation. His reasons for this advice are, 1st, that the hæmorrhage is usually venous, and ceases almost immediately on the trachea being opened, while in the patient's semi-asphyxiated condition the colour of the blood affords but little clue as to its origin. If the hæmorrhage be arterial, the vessel is usually of such small size that the bleeding can be checked by the application of nitrate of silver. And, 2d, blood which enters the bronchi does not, as is commonly supposed, occasion any serious consequences, but is expectorated as readily as the mucus which collects in the air-tubes, or the fluids which may be injected into them.—*Brit. and For. Med. Rev.*, from *Journ. des Con. Medico-Chirurg.* *October*, 1841.

---

*Nitrate of Silver in Gonorrhœa.*—Mr. Carmichael, of Dublin, has strenuously repudiated the practice attributed to him by Mr. Acton, of using injections of nitrate of silver, so strong as ten or twelve grains to the ounce of water. In Mr. C.'s published work on the venereal disease, he stigmatizes the above mode of treatment, as "a practice that cannot be too strongly deprecated." In a more recent publication—a lecture—he states that he has used the injection, but never in a greater degree of strength than one grain to one ounce—more frequently a quarter or half a grain to that quantity.—*Med. Gaz.*

---

[In the Dublin Journal for January, 1842, Dr. Graves has given a number of interesting cases of disease, offering different points of interest. His mode of looking at such clinical facts is always agreeable to us, and from the practical turn of most of our readers, we are satisfied that it is equally so to them, and shall continue our extracts.]

### *Arachnitis, with extensive tubercular disease of the lungs; both affections latent.*

A young policeman, apparently of strong constitution, and on active service up to the date of his present attack, was admitted on the tenth day of his illness into our fever wards. His surface was cold; his feet and hands blue; his pulse seventy, exceedingly weak; he lay in a listless state; he sometimes answered questions slowly, but rationally; but at other times paid no attention to what was said to him. He had complained of pain in the forehead the first ten days of his illness, but though the question was frequently put to him afterwards, he always expressed himself perfectly free from any pain or uneasiness in that part. During the day he was quiet, but towards evening he generally became delirious and violent, and on those occasions it was found necessary to apply the tight vest. His head was always cool; he had no contraction of the pupils, no increased pulsations of temporals or carotids, no suffusion of eyes, and no sweating of face or forehead. From the date of his admission until that of his death, which occurred sixteen days afterwards, he never exhibited the least febrile disturbance; his pulse fell down to sixty, was weak but regular; *his respirations always* perfectly natural, and he was never observed to cough. Though in the progress of his disease he got subsultus, jactitation, muttering, and complete insomnia, yet all this time the head was cool, and he presented no positive symptom of an active inflammatory process going on in the brain. Indeed the disease was so protracted, and the disturbance of the nervous system so similar to what frequently occurs in fever, uncomplicated with inflammation, that I pronounced his disease to be nervous fever. Four days previously to death, purging had been present.

*Post Mortem.*—The dura mater was quite healthy, but the arachnoid in several situations opaque and thickened. At the base of the brain, the nerves were all matted together by a thick yellow lymph, which extended from the optic commissure to the medulla oblongata, concealing from view all those parts which form the floor of the third ventricle, and also the origins of the third and sixth pairs of nerves. The arachnoid covering this lymph was thickened and opaque. The pia mater was much injected, and the substance of the brain was more vascular than natural, but in other respects normal.

The chest was next examined, and our investigation in this cavity disclosed what we were not prepared to expect. Both lungs were extensively studded with tubercles, and were in every part occupied either by phthisical abscesses or emphysema. The entire of both upper lobes was converted into abscesses, varying in size from that of a hen's egg to that of a Spanish nut, and communicating freely with one another. These abscesses were not of recent formation, for in every instance their walls were hard, thick, and cartilaginous, and some of the larger were traversed by blood-vessels. Most of them were full of puriform matter; in some, the contents were perfectly purulent, in others, pus mixed with blood, resembling the prune-juice sputa of pneumonia. The head (heart) was healthy; the ileum was healthy but the cœcum was inflamed, and presented many ulcers of a long irregular shape, extending through every structure down to the peritoneal coat, which, on the outside, presented no unusual appearance opposite these ulcers. Their edges were elevated, hard, and well defined.

[We cannot agree with Dr. Graves that this case was strictly latent; the affection of the lungs was marked by the disease of the brain, which rendered the respiration nearly natural. The cerebral disease was undoubtedly tuberculous arachnitis, that is, the acute hydrocephalus, as it is termed, which is so frequent in children. The disease has been so completely studied of late years that it is unaccountable that Dr. Graves should have overlooked it. It is particularly instructing, as it shows us very clearly what previous observation had confirmed, that the disease is sometimes almost as difficult to recognize as to cure.]

*Glossitis terminating in abscess.*

Idiopathic glossitis is an affection by no means common, and, I believe, it is even more rare to find it terminating in circumscribed abscess. For the above reasons I have thought it right to publish the following case.

Robert Anderson, æt. 30, was admitted into the Meath Hospital, complaining of pain in the tongue, with difficulty of swallowing, and indistinctness of articulation. The tongue was enlarged, particularly on the left side, about the centre of which a well defined tumour existed, hard and extremely painful to the touch. Pulse ninety, hard, and full. He had not taken any medicines that could have produced this affection. On the following day a soft spot was detected on the under surface of this hardness, which, on being punctured, gave exit to about a thimble full of thick, yellow, and very offensive pus. From this he got instantaneous relief,and left the hospital the same evening.

[The case of articular rheumatism is also curious.]

*Acute Articular Rheumatism, attended with pericarditis, and afterwards by symptoms of Delirium Tremens.*

A man named Reddy, æt. 27, was admitted into Meath Hospital, June 19, 1841. He was workman in the porter brewery of Messrs. Guiness, and was in the habit of consuming daily large quantities of their famous XX porter, besides whiskey. Three weeks before admission he was attacked with rheumatism in all the large joints, which when we saw him were swollen, red, and painful, the fingers of both hands were semi-flexed, and he could not bear them to be touched ; his countenance was dejected and expressive of intense suffering ; pulse 72, weak but regular ; heart's action normal ; profuse sweating ; inability to move in bed ; insomnia ; loss of appetite and thirst. He was bled and put on the use of calomel and opium ; the quantity of opium taken daily was *four* grains. The next day, 21st, pericarditis was detected, there was nothing remarkable in the signs ; the mercury and opium were continued ; cupping over the heart followed by blisters directed, and on the twenty-fifth day salivation set in ; the cardiac symptoms subsided, and the inflammation of the joints greatly disappeared. The quantity of calomel was diminished from twelve grains daily, combined with four grains of opium to three of the former with one-fourth of the latter every second day. On the 26th the rheumatism appeared much relieved, and the pulse was 88, soft and regular, yet there was something unusual about his appearance ; his countenance was excited and his eyes bright, and on inquiry we ascertained that he had slept none during the night, and that he had raved the whole time, occasionally shouting and singing. On the 27th he was much worse, he lay quite prostrated on the bed, the upper part of his body was covered with a profuse perspiration, he had twitching of all the muscles of the face, subsultus, and tremor of the lower limbs, he slept none, but raved all night, and about three o'clock, A.M. got out of bed, and endeavoured to break through a door into the adjoining ward. His tongue was dry and unsteady when protruded, he answered questions, however, rationally, and said he had *no headach ;* pulse 116, very weak.

He was now ordered one grain of opium, in the form of pill, every fourth hour, and four ounces of wine in the day.

On the 28th the report states that he fell asleep after the third pill (about eleven o'clock,) and did not waken for six or seven hours, when he again commenced shouting and singing, but soon became quiet, and at eight o'clock the following day the tremors and had greatly diminished ; his countenance was vastly improved, skin cool, tongue steady when protruded, but dry and furred, and his intellect restored. It was found necessary to increase the wine from four to sixteen ounces since the 27th.

On the 28th all the symptoms of delirium tremens had vanished, he was free from headach, his skin cool, tongue moist and no thirst, and the pain in the joints nearly gone.

The wine and opium were now both diminished gradually, and in ten days after he was discharged perfectly cured.

The complication of delirium tremens with acute rheumatism is not by any means common, and it is remarkable that in this case the first symptoms of the affection manifested themselves the day after the quantity of opium was diminished. Can we explain this by supposing that the opium acted as a stimulant, and that being stopped suddenly it produced the same train of symptoms that usually follow the leaving off of any strong stimulant that had previously been largely indulged in.

This explanation may seem at first plausible, but we know from experience that when opium acts *beneficially*, as a medical agent, it seldom produces any of the bad consequences that follow its exhibition in a healthy state of the body, an illustration of which this case affords, for we find that it neither occasioned headach, heat of the skin, furred tongue, thirst, contracted pupil, or acceleration of the pulse. We must, therefore, look upon the circumstance as a mere coincidence, and we can easily comprehend how delirium tremens might occur in a patient of intemperate habits during the course of a painful illness, by which he was much reduced and worn down.

Opium has lately been much employed in the treatment of acute articular rheumatism, and we are indebted to Dr. Corrigan for an excellent paper on this subject. The practice is, however, by no means new, for opium has long been employed for this purpose in America, and I find the celebrated Doctor Pearson of London used it in preference to all other remedies.

In the third volume of the Medical Gazette there is an account of a discussion at the Westminster Medical Society, upon the treatment of acute rheumatism, and Dr. Gilbert Burnett is reported to have said, that "he was a pupil of Dr. Pearson's, but had always heard him speak of calomel with absolute detestation, *while he administered opium in all cases of rheumatism almost to the exclusion of any other means.*" This does not, however, detract in the least from the merit of Dr. Corrigan's paper, for we are indebted to it for the revival, if not for the origin of a very excellent mode of treatment, and one that will be found, in many cases, to succeed admirably.

---

*Ultimate results of the operation for Strabismus.*—[Frederic Beverly Dixon, Esq., of Norwich, in a letter to the editors of the London Medical Gazette, of November 19th, 1841, presents the results of forty-one cases of strabismus convergens, operated upon in November, 1840, and inspected in October last. The object of the writer is to add to the list of well observed cases, in order to test the tendency to abduction of the eye, which occurred within ten months after all the operations of Mr. Barker, reported in the same Journal in August last. We give the following condensed view of the results of Mr. Dixon:

Both pupils remained perfectly central in thirty-one cases; twenty-three of which were inspected by the surgeon, and eight were reported by the friends of the patients. The eye subjected to the operation rested perfectly straight, while the untouched eye presented a slight obliquity in five cases. There was complete return of the deformity, after a time, in three cases. The pupil became abducted, so as to produce a leer in two cases. In most of the successful and partially successful cases, vision was improved.]

---

*Nitrate of Silver in Gonorrhœa.*—[Some conversation took place in the Westminster Medical Society, on the 13th of November, on the subject of injections in Gonorrhœa. We extract the following observations from the report in the London Lancet of the 20th.]

The President (Mr. H. J. Johnson) in old gleets had used the injection as strong as a drachm of the nitrate to an ounce of water, with good effect: much inflammation followed, and a cure was effected in several cases.

Mr. Harding had used ten grains of the nitrate to an ounce of water with benefit in these cases.

Mr. Streeter had found in old gleety discharges that a solution of sulphate of copper, when used with care and judgment, was a most efficacious remedy.

Mr. Acton did not employ the solution of the nitrate of silver of the strength mentioned by either of the speakers. All the good effects of the salt were, in his opinion, to be produced by solutions of two grains in eight ounces of distilled water: used in these quantities, and with the recommendations given in his work, he believed that few gleets would fail of being cured; he had never with this treatment had to regret the intense pain or inflammation which other gentlemen had spoken of. His object was not to produce inflammation of the canal by means of the salt; he believed that it should be the surgeon's intention to alter the action of the part, at least he found that was all we required.

---

*Report of the paper of J. R. Martin, Esq., formerly Surgeon of the Native Hospital, Calcutta, on Injections of Iodine in Hydrocele, and the subsequent debate in the Royal Medical and Chirurgical Society of London.*—In consequence of an accident of a serious nature which occurred several years ago to a native in the Calcutta Hospital, upon whom the usual mode of treating hydrocele was adopted, viz. that by port wine injection, the author, in the next case that presented itself, substituted tincture of iodine for the wine. Two common urethra syringefuls were injected, of a mixture made in the proportion of one drachm of the tincture to three drachms of water. Acute pain and faintness followed, which were relieved by the recumbent posture, and the injection was *retained:* the scrotum being moved about so as to bring the fluid into free contact with the vaginal cavity. In five days the patient was discharged cured, scarcely any other treatment having been necessary.

From the time of the occurrence of this case, in March 1832, to the end of 1839, 2393 cases were operated upon in the native hospital, under the orders of the author, in the manner above described: some cases only, in which the tumor was of a very great size, having required two syringefuls of the injection.

The author, after entering into the details of many of the cases in which he employed the above treatment, sums up its advantages as follows—

1. That it is far more simple and easy of performance than any operation before employed.

2. That no serum has in any case been reproduced requiring a second tapping.

3. That little care is required in the after treatment.

4. That failures are one per cent.

5. That the operation is free from all danger of infiltration of the soctrum, from the quantity of injection being so small, and from its being retained in the tunica vaginalis.

Dr. Mayo called on Mr. Stanley to favor the Society with his opinion respecting the danger of the injected fluid infiltrating into the cellular tissue.

Mr. Stanley said that, he had heard, with some surprise, the statement made in the paper respecting the frequency of that occurrence. In the great number of cases of hydrocele that had been injected during the last five and twenty years at St. Bartholomew's Hospital, he had not known the accident occur more than three times at the most : of two cases he had a distinct recollection, of a third he was less certain. He did not think there was anything in the author's plan of treatment particularly calculated to render that accident less frequent than it was in the ordinary plan.

Mr. Macilwain said, he thought the accident more frequent than Mr. Stanley did: he had seen three cases which had occurred not to himself, but to practitioners who had called him in, in consultation; but he believed that in every case the accident was to be ascribed either to some fault in the patient, or to some want of caution in the operator. It was thought a very trivial operation, and one who had often performed it was apt to neglect those little cautions which were necessary for its security. The rule which he believed was always to be observed, he had heard laid down by Mr. Earle, to whom he was pretty sure no accident had ever happened: namely, to take care, in withdrawing the trocar, to press forward, at the same time, the canula. He did not believe that, in regard to the frequency of the accident of infiltration, any difference at all could depend on the kind or quantity of the fluid injected.

Mr. Perry mentioned a case, one of those he presumed which Mr. Stanley had also seen, in which the infiltration had taken place through the fault of the patient. That case proved, however, that the danger arising from infiltration was not so great as it was represented to be; for by slightly enlarging the orifice through which the trocar had been passed, and injecting warm water into the cellular tissue, all important injury was prevented. His own experience fully confirmed the rarity of these accidents in England.

Mr. Stanley thought the question of chief interest connected with the paper was, not the prevention of infiltration, to which the author's plan of treatment had no particular relation, but the certainty with which the injection of iodine seemed to produce the desirable result. If it were certain that (as the great number of cases by the author seemed to prove) the inflammation thus produced was both set up more speedily, and subsided with proportional speed, so that the disease was both more quickly and more certainly cured than by any other kind of injection, he thought a practical point of much importance was established.

Mr. Macilwain held that the ordinary plan of treatment by port wine injections was, with proper care, as certain as any remedy could be. If it often failed, it was only because attention enough was not paid to accompanying circumstances. He had heard Mr. Abernethy say, and it was quite true, that for the cure there was a certain degree and kind of imflammation to be produced, and no other, and the production and maintenance of this depended on many circumstances besides the mere injection. He had treated a vast number of cases of this disease, and many of them under most unfavorable circumstances, and the result had been so nearly invariably successful that he was not disposed to abandon the use of wine injection for any other that had been proposed.

Mr. Arnott thought it was much to be regretted that the author had prefaced his paper with remarks about accidents and errors of other surgeons, of which he had not himself been witness, and of which the Society was in no wise called upon to take notice. He had seen some cases in which serious infiltration of the cellular tissue of the soctrum had taken place after the injection, but quite independent of the passage of the injected fluid into it, and he thought it probable that some of the cases that had been referred to as examples of infiltration from accident or carelessness were of this kind. However the question to be considered was, as Mr. Stanley said, whether the iodine injection were better adapted to produce the requisite degree of inflammation than any other. The author's experience would seem to prove, beyond a doubt, that it was. But there was a circumstance overlooked: viz. the greater tendency to inflammation which it was well known there was in the inhabitants of tropical climates: it was quite possible that this plan might be very successful in India, and yet fail in Europe. However, he believed, for his own part, that if tried in this country it would be found to succeed better than any other method.

Mr. Charles Hawkins mentioned four cases which he had seen thus treated, but in which the results were not encouraging.

Mr. Perry begged to ask Mr. Acton whether he knew if there were foundation for the reported opinion of M. Ricord on the operation.

Mr. Acton said that though he had been attending M. Ricord's practice at the time when the accident was said to have happened to him, he did not believe that M. R. had ever thrust the trocar into the testicle. He believed that it had been done by a surgeon in Paris, but it was not M. Ricord. He had seen both M. Ricord and M. Velpeau frequently employ the iodine injection in this disease: he had never seen it produce any ill effect, and his impression was, that it was to be preferred before the use of wine or any other irritant. It was especially preferable to the French mode of injecting wine: for it was in that country customary to make up for the usual weakness of the wine by injecting it hot into the soctrum, a practice which he believed was never adopted in this couutry, and which he thought might have led to some of the evils attributed to the infiltration of the injection. He had not in any case seen that excessive pain produced which had been spoken of as one of the effects peculiar to the iodine injection.—*London Medical Gaz. Nov.* 19, 1841.

---

*The Medical Examiner* is published every Saturday by *J. C. Turnpenny*, N. E. corner of Spruce and Tenth streets. Terms—*three dollars* a year for one copy, or *five dollars* for two copies sent to the same post office, in all cases in advance. Postage the same as on newspapers. Letters to be addressed,—post-paid,—to the Editors of the Medical Examiner, Philadelphia.
*Reynell Coates, M. D.*, Acting Editor.
*J. B. Biddle, M. D.*, and *W. W. Gerhard, M. D.*, Co-editors.
*J. B. Biddle, M. D.*, Proprietor.

